# Training Bioethics Professionals in AI Ethics: A Framework

**DOI:** 10.1017/jme.2025.57

**Published:** 2025

**Authors:** Etienne Aucouturier, Alexei Grinbaum

**Affiliations:** CEA-Saclay, France

**Keywords:** AI Ethics, Training, Bioethics, Artificial intelligence, Research Ethics Committees

## Abstract

We present a training module in AI ethics designed to prepare a broad group of professionals to recognize and address potential ethical challenges of AI applications in healthcare. Training materials include a two-page checklist, a brief glossary, and three practical case studies. While we have developed and applied this framework for training Research Ethics Committee members in France and South Africa, it can also be helpful in university courses ranging from public health and healthcare law to biomedical engineering and applied ethics.

## Introduction

The arrival en masse of tools and technologies using artificial intelligence (AI) in all domains of healthcare underscores the need to swiftly adapt bioethics curricula to include relevant elements of AI ethics. Students as well as professors from different disciplines (biology, medicine, law, philosophy, public administration, etc.) need to be trained to recognize and address potential challenges and opportunities of AI in healthcare. While this need encompasses all aspects of teaching healthcare law and ethics, it is also a significant concern for the evaluation of research projects performed by institutional review boards (IRBs), *comités de protection des personnes* (CPPs), and other types of Research Ethics Committees (collectively referred to as RECs).

Historically, the majority of projects evaluated by RECs dealt with bioethical concerns or with research protocols involving human participants. However, research projects developing AI systems or using AI tools require a new kind of expertise. REC members across Europe and internationally express concerns about their capacity to address issues at the intersection of biomedical ethics and AI ethics.^1^ There is also a significant risk that poor understanding of the underlying AI research may lead to inadequate evaluation by REC members, who tend to overestimate or to underestimate the importance of ethical issues.^2^ Researchers with different professional backgrounds (biologists, biophysicists, biochemists, lawyers, psychologists, linguists, sociologists, philosophers, etc.) dealing with bioethics law and regulation need to develop novel evaluation skills and at least a basic understanding of AI ethical issues.

To address this challenge, we have developed a training framework designed to educate university professors, researchers, students, and REC members on ethical issues associated with AI in healthcare. This framework can be incorporated into university courses ranging from public health and healthcare law to biomedical engineering or applied ethics. Here, we provide guidance on running a training session, describe educational materials, and discuss the lessons learned from running several training sessions in the Horizon Europe iRECS project.

In Section 2, we discuss the training needs and barriers for bioethics professionals facing complex ethical issues in computer science. In Section 3, we present the methodology and essential elements of our framework: the checklist, the glossary, and the mock ethics review exercises. In Section 4, we discuss lessons learned in iRECS.

## Analysis of Needs

Scientific literature on training for bioethics professionals has rarely, if ever, systematically addressed AI ethics. The 66 empirical studies and 106 ethics courses listed in a 2017 review include no targeted training on AI in healthcare.^3^ To take an example, an interesting reflective account of the implementation of REC training in Croatia emphasized the need for continuous training to enhance the knowledge of REC members,^4^ however the focus was on medical ethics and hospital IRBs rather than on emerging ethical issues in research. This patient-centered approach in bioethics remains to be reconciled with AI ethics.

Our approach is derived from the broader regulatory frameworks in AI ethics, including the High-Level Expert Group on AI (AI HLEG) Assessment List for Trustworthy AI (ALTAI),^5^ which is also referenced in recital 27 of the EU AI Act.^6^ These documents provide frameworks for assessing ethical issues in AI research, although they often appear too broad and complex to be directly operational for evaluating a particular AI application or a research project. Additionally, Chapter 8 of the *EU Ethics Appraisal Scheme* ^7^ lists AI-related ethical concerns on the basis of the AI HLEG but does not provide guidance on how to evaluate the answers provided by the applicant. The need for training REC members is evident, even if the rise of such concerns is not new and can be traced to the mid-2000s.^8^

Two problems need to be addressed in this regard: first, reducing the complexity of the existing guidelines to make them applicable and operational; second, improving the availability of experts capable of applying these guidelines. These problems have become more pressing with the emergence of specific AI-related ethical concerns. These include questions of bias, transparency, explainability, overreliance, or AI-related biosafety. Today, such issues are not systematically evaluated by RECs, with some AI research in healthcare falling outside traditional bioethical oversight due to regulatory gray zones.^9^ Consent and privacy remain focal points in REC evaluations, especially as AI projects introduce new challenges related to the future use of data and the limitations of traditional consent models.^10^

To improve ethics review practices, new models have been proposed. The notion of “ethics by design”^11^ is based on the idea of respecting fundamental values when designing a technical system. Ethics by design can be understood within different theoretical and methodological frameworks,^12^ most notably “value-sensitive design” and “technology assessment.”^13^ More recently, Bernstein et al. advocated for the establishment of ethics and society review boards alongside traditional RECs.^14^ Another model, proposed by McLennan et al., is the embedded ethics approach,^15^ where ethics advisors are involved throughout the research process to guide decision-making from planning to implementation.

The ex-ante model of ethics review, traditionally used by RECs, is often inadequate for AI projects, as many ethical issues only become apparent later during project development and implementation. Ongoing normative evaluation^16^ is necessary to manage the complex ethical challenges posed by AI. However, for these models to be effective, RECs must be equipped with sufficient resources and expertise. Without proper resources, expanding the role of RECs in AI research risks overburdening them, potentially compromising the quality of ethics reviews.^17^ Moreover, a proportional approach to ethics governance, where the stringency of review is matched to the level of risk and technical feasibility, as suggested by the EU AI Act, may provide a more sustainable model for ethics oversight.^18^

To move beyond the traditional ex-ante model, RECs need to include AI ethics professionals who will ensure follow-up on REC recommendations and remain in touch with the project team during implementation. This implies that RECs become, at least partly, somewhat like Digital Ethics Committees (DECs) providing continuous oversight throughout the lifecycle of AI projects. To move beyond one-time compliance checks and adopt the “ethics-by-design” approach, members of RECs should enhance their knowledge and expertise and develop a mechanism to consult with AI experts whenever necessary.

## Training Materials

To develop an effective training framework, we use the ADDIE methodology (for Analysis, Design, Development, Implementation, and Evaluation), which was originally conceived in the mid-1970s.^19^ This well-established method allows for a structured approach to both instructional and training design, incorporating feedback loops that enable ongoing adjustments throughout the training process.

The first ADDIE phase, Analysis, began in late 2022 and culminated in a report in early 2023. We formulated an analytical framework and developed a blueprint for expert consultations aimed at identifying specific training needs.^20^ Based on this, we designed two formats for training modules: a brief awareness session (1 hour lecture) and a half-day module (3 hours). We consulted with iRECS experts, many of whom are REC members, to inform the content of these sessions. This approach allowed us to incorporate direct input and narratives regarding the challenges faced by REC members. Expert consultations were guided by a structured set of questions, contributing to an analytical framework for identifying gaps in ethics review procedures. These questions were organized into two distinct categories: the first one focused on informing the development of training materials and awareness-raising initiatives, while the second one addressed existing deficiencies in ethics review processes. This dual approach ensured a comprehensive assessment of both the educational and procedural needs, facilitating the design of targeted interventions for enhancing ethics oversight across various regulatory environments.

Based on this analysis, we proceeded with the Design phase in early 2023, where we created and tested the initial training materials. These materials were further developed by early 2024. We began the Implementation phase in June 2024, running the Evaluation phase in parallel. The feedback received from REC members who participated in trainings allowed us to make adjustments to the framework, as discussed in Section 6.

Our training framework consists of three main components (see Appendix): a checklist, a glossary, and a set of three mock AI research projects in health and healthcare. These exercises are intended to serve as a pedagogical resource allowing the trainees to apply the checklist and conduct an evaluation as close to real-life situations as possible.

### The Checklist

The checklist includes eight chapters: Role of AI systems in the project; Explainability and reproducibility; Data; Bias and fairness; Cybersecurity and biosecurity; Human oversight and accountability; Beneficence, non-maleficence, and human autonomy; and Socioeconomic and environmental impact. Each chapter is further divided into checkpoints, similar to the HLEG ALTAI approach.

Information on the role of AI systems in healthcare projects is critical, as it establishes the scope and context of their application. Understanding whether the project utilizes, develops, or integrates third-party AI systems informs stakeholders about associated risks, regulatory requirements, and operational responsibilities. Clarity regarding the Technology Readiness Level (TRL) helps assess the project’s maturity and readiness for clinical use, ensuring that safety and efficacy standards are met, particularly when the system is intended for vulnerable populations.

Explainability and reproducibility are key ethical considerations in AI, especially in healthcare, where transparency impacts trust and accountability. The ability to explain how an AI system reaches conclusions fosters patient and doctor confidence and facilitates informed decision-making. Ensuring that outputs can be reproduced is essential for validating AI systems, as reproducibility underpins scientific integrity.

Data management is also a cornerstone of AI deployment in health and healthcare research, directly impacting patient privacy, consent, and data integrity. Proper handling of sensitive data ensures compliance with legal frameworks, such as the General Data Protection Regulation (GDPR). By focusing on data collection procedures and quality, the project mitigates risks associated with data misuse and bias. This careful approach not only safeguards individuals’ rights but also strengthens the overall credibility of the AI system, ensuring that it serves its intended purpose without compromising ethical standards.

Addressing bias and fairness is imperative to prevent discrimination and ensure equitable treatment across diverse populations. Analyzing the balance of datasets and the potential impact of AI outputs on various demographic groups is essential. By implementing countermeasures against biases, such as using synthetic data, and ensuring that healthcare access is not adversely affected, the research project actively works toward minimizing health disparities, reinforcing the moral obligation to promote equity in healthcare delivery.^21^

Cybersecurity considerations are increasingly relevant as AI systems become integral to healthcare. These measures protect sensitive patient data and ensure the integrity of AI operations against malicious attacks. Additionally, there is a growing concern about biosecurity and the misuse of AI in creating biological or chemical warfare weapons, which poses a significant threat to global security.^22^ By addressing vulnerabilities, the project prevents potential harm to patients and healthcare providers. Emphasizing robust security protocols enhances patient safety and fortifies trust in AI technologies, which is critical for widespread acceptance and successful implementation in clinical settings.

Human oversight and accountability are essential for the use of AI in healthcare, where decisions can significantly impact patient outcomes. Establishing clear supervisory roles ensures that AI systems operate within a framework of human values and ethical considerations. This oversight is critical for addressing unforeseen outcomes and assigning responsibility in case of errors or harm. By incorporating AI ethics experts and fostering a culture of accountability, the project creates a framework that prioritizes patient welfare and promotes trust among stakeholders.

The bioethical principles of beneficence and non-maleficence are foundational and particularly relevant when integrating AI systems. Ensuring that patients are informed about the benefits and limitations of AI fosters informed consent and respects patient autonomy. Furthermore, the ability of the AI system to support rather than undermine healthcare professionals’ decision-making is crucial for maintaining high standards of care. By actively involving patient representatives, a project aligns AI applications with the needs and values of the communities they serve.

Finally, assessing the socioeconomic and environmental impacts of AI deployment in healthcare helps us to understand its broader implications. The need for new skills underscores the importance of preparing healthcare practitioners for working with AI systems. Additionally, addressing the risks of overreliance on AI ensures that practitioners maintain their competencies. By evaluating potential environmental impacts and implementing mitigation strategies, a research project also fosters a sustainable approach to healthcare innovation that aligns with ethical and social responsibilities.

### The Glossary

The glossary includes twelve entries selected to enable the most basic understanding of AI: artificial intelligence system, machine learning, fine-tuning, alignment, explainability, reproducibility, hallucination, bias, emergent capability, adversarial attack, synthetic data, and ethics by design. These twelve terms provide a minimal set required for navigating the challenges and complexities of AI systems in healthcare, highlighting the importance of transparency, security, and responsible oversight.

### The Exercises

The three mock research projects cover all sections of our checklist. In each case we provide the following instructions:

1. *Using the “Ethics of AI in Healthcare” checklist, identify major ethical issues in this project.*
2. *Select serious ethical concerns and formulate recommendations.*

The first instruction aims to engage the trainees’ analytical skills by encouraging them to screen the mock project while reviewing the entire checklist. The second aims to stimulate their critical thinking, by asking them to evaluate the comparative seriousness of ethical concerns. What is crucial for a reviewer is that they identify the truly limiting factors with a serious potential to endanger health, safety, or fundamental rights.

The first mock review exercise is focused on preventing recurrences of Major Depressive Disorder (MDD). Ethical issues primarily arise around beneficence, non-maleficence, and respect for human autonomy. While the AI-based coaching system aims to improve the quality of life for MDD patients, there is a need to ensure that the technology does not cause harm, either by overestimating the efficacy of AI interventions or by undermining the patient’s autonomy in decision-making. Human oversight and accountability rank second in the results of our test training sessions, as the combination of unsupervised machine learning and clinical practice requires clear guidelines for mental health practitioners to remain in control of patient care. It is essential that mental health professionals do not become overly dependent on AI recommendations, and that accountability for decisions remains with human practitioners. Lastly, data privacy is a major concern. Ensuring that patient data is protected throughout the data collection, analysis, and feedback processes is crucial for safeguarding patient trust and meeting ethical standards.

The second exercise concerns AI-driven drug design. It raises several ethical concerns, with biosecurity ranking as the foremost issue. The generative AI model, designed to predict viral mutations and simulate hypothetical virus variants, could be vulnerable to misuse. There is a risk that these AI-generated datasets might be exploited for harmful purposes, making robust biosecurity measures essential to prevent the accidental or malicious development of bioweapons. Secondly, explainability is a significant concern, as the complexity of deep learning models and their iterative training on vast, heterogeneous biobank data may make it difficult to fully understand or audit the model’s decision-making processes. This lack of transparency could hinder the ability of scientists and regulators to ensure the system’s reliability and safety. Finally, the project also raises biosafety and socioeconomic concerns. The global accessibility and affordability of the resulting drugs need to be considered, as this project aims to create commercially viable treatments.

The third exercise focuses on enhancing breast cancer diagnostics using AI across a network of hospitals. Ethical considerations here include ensuring robust data protection, maintaining patient privacy, and guarding against potential overreliance on AI for critical medical decisions. One major issue is the potential overreliance on AI, which could lead to a loss of competence among healthcare practitioners. Additionally, biases and fairness are critical concerns, as it is unclear whether the system will be trained on sufficiently diverse data to be relevant for patients worldwide, raising questions about global accessibility and utility for a commercial product. Another significant issue is explainability, as the decentralized nature of federated learning may complicate efforts to fully audit or explain the model’s decisions. Secondary ethical concerns include cybersecurity risks, such as malicious participants corrupting the model with poisoned data or adversarial attacks, and privacy risks, where personal data could be indirectly exposed during model updates despite the lack of centralized data storage.

### Running a Training Session

We designed these materials to support an interactive training session lasting three hours, organized into three chronological segments of approximately one hour each: (1) a plenary presentation of the key technical and ethical aspects of artificial intelligence with a focus on health and healthcare issues; (2) a group exercise segment involving mock ethics reviews; and (3) sharing findings and discussing the results with other group members. We provided participants in the training with the checklist and glossary in advance, allowing them to familiarize themselves with the terminology and specific ethical issues related to AI in health and healthcare.

## Lessons Learned

The use of checklists in ethics appraisals has both advantages and drawbacks and is subject of ongoing debate.^23^ On the one hand, checklists help ensure that all necessary ethical considerations are systematically covered, and no important issues are overlooked, leading to more complete evaluations. They can also streamline the review process, making it quicker and easier for reviewers to identify ethical issues. On the other hand, critics argue that checklists can oversimplify complex ethical issues, reducing them to a “ticking boxes” exercise that may discourage deeper critical thinking.^24^ They may also lack the flexibility needed to adapt ethical reflection to the unique nuances of each research project, potentially resulting in inadequate ethical assessments. Additionally, relying on a checklist for ethics evaluation can create a false impression of ethics “clearance” if all boxes have been checked. Ultimately, the effectiveness of checklists in ethics appraisals depends on how they are used. They can be valuable tools if integrated into a reflective ethical evaluation performed by a thoughtful expert. They can also help a less experienced researcher to ask the right questions in an exhaustive way, if the design of the checklist underscores context sensitivity and the need for critical assessment of the seriousness and complexity of potential ethical concerns.

In our training, we explicitly told the participants about the potential drawbacks of using checklists. The goal was to warn trainees about the pitfalls of oversimplification and inflexibility, while maximizing the intellectual benefits of checklists. Hence the focus of the mock review exercises was put on selecting serious and complex ethical issues among the plethora of potential concerns mentioned in the checklist. According to the qualitative feedback we received from our training sessions in France and South Africa, a detailed checklist seems particularly well-suited to contexts where the technical knowledge is complex or evolving, such as in the case of AI.

The pedagogical strategies for structuring the training framework are grounded in both the psychological approach by the trainers and the technical competency required of them to ensure effective learning by REC members. On the psychological side, it is important to approach the target audience with humility to offer complementary expertise to the already-existing biomedical expertise in RECs. It is also important that the trainers handle with openness and professionalism the situations where the technical expertise of trainees exceeds their own. On the more pragmatic side, the insights include the following three points.

First, it is necessary to strike a balance between technical details and the criterion of accessibility. Choosing the right language is not an easy task, for example, in explaining the functioning of large language models (LLMs). Similarly, explaining the importance of synthetic data for training AI models may require an explanation of why this type of non-real data is crucial for building successful real-world applications of AI systems across many healthcare sectors. Trainers must be prepared to provide clear and faithful answers to technical questions in a language that is relatively simple to understand.

Second, beyond technical fluency, trainers must possess a robust understanding of the broader philosophical and ethical issues related to AI. Ideally, trainers should have expertise in the philosophy and history of AI. This allows them to frame ethical discussions within the appropriate context, ensuring that ethical concerns are directly related to the core scientific concepts and technical components of AI systems.

Third, trainers must emphasize the velocity of change in AI and the importance of ongoing self-education. For example, the relevance of ethical concerns related to the hallucinations produced by LLMs was higher in 2021–2022 than in the current generation of frontier models, yet many REC members put hallucinations as the most serious concern of models with more advanced alignment. Showing how quickly the complexity and seriousness of ethical concerns in AI ethics evolve in time is an integral part of training AI ethics evaluators.

## Conclusion

Our training framework on AI ethics in healthcare is a first step in addressing the challenges associated with the growing ubiquity of AI across biomedical research. While it can be used by professionals from many related disciplines around the globe, this framework is based on the European Union’s normative guidelines. Adapting it to the US or other jurisdictions will require relatively minor adjustments, as the key areas of concern remain the same. Above all, this framework is built to meet the need for interdisciplinary training in AI ethics and bioethics, which is currently present in all national regulatory contexts.

The training sessions we conducted in France and South Africa also highlighted challenges. The diversity of REC members’ backgrounds implies that technical knowledge about AI will not be equally appreciated by all trainees. Additionally, rapid advancements in AI technology necessitate ongoing updates to the training materials. We expect that the attached set of materials will remain “future-proof” as the newly drafted EU General-purpose AI code of conduct^25^ aims to be, for at least five years.

In conclusion, this training framework can be seen as a pedagogical resource for university courses across many related disciplines or schools. It also provides a foundation for RECs to improve the quality of ethics review of AI projects. By equipping REC members with key knowledge and the necessary skills, we provide support for informed decision-making during project evaluation.

## Ethics of AI in Healthcare: A Checklist for Research Ethics Committees

1. Role of AI systems in the project
  - Does the project use an AI system, develop an AI system, or both?
  - If an AI system is developed in the project, up to which Technology Readiness Level will it be developed (research/industrial prototype/scalable commercial product)? Are compliance checks and certification included?
  - If a third-party AI system is used in the project, is it a commercial solution or a research prototype? Is it certified (CE or FDA)?
  - If a third-party AI system is used in the project, is it operated by health professionals or non-medical staff?
  - Will the AI system be used by, or operated for, patients, healthy subjects, and/or vulnerable groups?
2. Explainability and reproducibility
  - Does the AI system use supervised learning for training or for fine-tuning? If yes, is the selection of the annotators adequately explained? Are they fairly compensated for their work? Are there measures to guarantee the well-being of the annotators in case they are exposed to toxic content?
  - Does the AI system use non-supervised (self-supervised) learning? Are there measures to ensure explicability? Is there a procedure for improved explicability of the AI system’s outputs with a low confidence score? Is there a procedure for explaining and acting upon incidental or unexpected findings?
  - Does the project involve generative AI? Is the generative AI model used in the project proprietary or open-source? Is the choice of the model justified?
  - Are there verification and validation methods to evaluate reproducibility of the outputs provided by the AI system?
3. Data
  - What datasets are used for training and/or for fine-tuning the AI system? Are they public or proprietary? Will a new dataset be built?
  - What data collection and storage procedures are put in place for building the dataset? Is the data minimization principle respected? Is financial or other compensation offered in exchange for data?
  - Is data personal or sensitive under GDPR? Is it anonymized or pseudonymized? Is there an appropriate consent procedure mentioning the use of data for training AI systems with an explicitly specified purpose? Is it possible to allow persons to retract or delete their data from the dataset?
  - Are privacy-by-design technical measures considered?
  - Is the dataset synthetic or generated? How was it generated? What evaluations, bias measures, and documentation on synthetic data have been provided?
  - Has an effort been made to select high-quality data for training? How is data quality measured?
  - Is the dataset open and publicly available?
4. Bias and fairness
  - Is the dataset balanced with regard to relevant categories, e.g., sex or geographic representation?
  - Is the use of the AI system likely to result in discrimination against certain categories of persons?
  - If generative AI is used in the project, are there countermeasures to avoid hallucinations (plausible but incorrect outputs)?
  - Does the use of the AI system make access to healthcare more costly or less available for certain categories of persons? Are accessibility measures included?
5. Cybersecurity and biosecurity
  - Does the AI system implement measures against adversarial attacks or hacking?
  - What robustness measures are put in place in machine learning?
  - Can the AI system be misused? Is misuse likely to occur?
  - How are false positives and false negatives identified, traced, and analyzed?
6. Human oversight and accountability
  - Does the AI system operate under human supervision? Is supervision occasional or permanent? What control powers does the supervisor have?
  - Does the system inform the user and/or the operator about unusual or unintended findings?
  - Is there a procedure to assign responsibility in the event that the AI system causes damage?
  - Are there mechanisms to facilitate auditability and traceability of the AI system’s outputs?
  - Does the project include AI ethics experts or an Ethics Advisory Board with a mandate to oversee the development of the AI system?
7. Beneficence, non-maleficence, and human autonomy
  - Are patients, users, and medical staff informed of the purpose, benefits, and limitations of the AI system? Is this information provided in a clear and comprehensible way?
  - Does the AI system influence health professionals involved in the project? Can it subvert or impair their decision-making?
  - Does the AI system interact directly with patients? Does it impair patients’ autonomy?
  - Are there specific risk mitigation measures and measures to avoid putting patients at risk?
  - Are patient representatives consulted or involved?
  - If the project includes a randomized clinical trial, will patients in the control group have the chance to benefit from the use of the AI system?
8. Socioeconomic and environmental impact
  - Does using the AI system require new skills or competences for health practitioners? Is training included?
  - Are there measures to prevent overreliance of healthcare practitioners on the advice given by the AI system?
  - Are there measures to ensure that the use of the AI system will not result in the loss of competence of healthcare practitioners?
  - Is there a risk that the outputs of the AI system may lead to increased human or environmental toxicity? Are there mitigation measures against biosafety risks?

## Ethics of AI in Health and Healthcare: *Glossary*

**Artificial intelligence system:** in the narrow sense (excluding deterministic “expert systems”), a machine learning system designed to operate autonomously, learning from a corpus of data, demonstrating adaptability to different inputs and producing outputs (such as predictions, recommendations, decisions and other content).

**Machine learning:** automatic process by which information is generated in the form of mathematical correlations from a training dataset. Types of machine learning include reinforcement learning, supervised learning (using human annotations) and self-supervised or unsupervised learning (without human annotations). The result of machine learning is called an **AI model**. An AI model can be subjected to **fine-tuning** and **alignment**. When combined with a user interface, an AI model trained on a large corpus of data to perform a variety of tasks becomes a **general-purpose AI system**.

**Fine-tuning:** the process of tailoring an AI model trained on a large dataset to perform specific tasks, by refining its training on a specialized corpus of data.

**Alignment:** design and application of filters and control systems to prevent undesirable AI system behavior.

**Explainability**: the ability to provide a textual or visual explanation of the output provided by the AI system, which enables the user to achieve a satisfactory understanding of the underlying causes that led to this output.

**Reproducibility:** possibility of retrieving the same result after multiple executions of the AI system.

**Hallucination:** production by an AI system of plausible but false or unreal outputs.

**Bias:** distortions that occur when AI systems are trained on non-representative datasets, producing false or discriminatory results which can result in a loss of user confidence.

**Emergent capability:** an AI system’s behavior, perceived by the user, which emerges from its training without any explicit intention on the part of the designer.

**Adversarial attack:** an attack involving the injection of corrupted data or malicious inputs, designed to cause errors or induce undesirable behavior in the AI system.

**Synthetic data:** simulated data sets produced by a large-scale AI system (that can itself be trained on a set of authentic or synthetic data) with the aim of training a smaller-scale AI system.

**Ethics by design:** a methodology for analyzing, as early as the design phase of an AI system, the technological choices likely to give rise to ethical tensions. It aims to translate ethical principles into operational measures, while adapting them to evolving standards. It also includes ongoing evaluation of these measures in realistic use cases.

## Mock Review Exercises

### Prevention of Major Depressive Disorder Recurrences

The overall aim of this project is to combine the most advanced AI technologies with a socio-psychological approach to develop a coaching system for improving the quality of life at home of patients who suffer from major depressive disorder (MDD), also known as clinical depression. The system aims at detecting and managing MDD-related issues and at avoiding MDD recurrences.

Major depressive disorder (MDD) is among the leading causes of disability worldwide largely due to its highly chronic and recurrent nature. The risk of recurrence after a first major depressive episode is about 50% and it increases with subsequent episodes. After the primary hospital intervention, most MDD patients are sent back home for long-term treatment, making the disease comparable to a chronic condition. A decrease in the adherence to treatment may occur, compromising therapeutic efficacy.

This project will develop a patient coaching mobile app based on machine learning, with the objective of helping patients to follow their treatment:

- The app will rely on predictive models based on both retrospective and prospective data (clinical data, data from unobtrusive environmental and wearable sensors, data from social media and questionnaires in the app). Collected data will be made available to mental health practitioners.
- Unsupervised machine learning models will be combined with clinical practice.
- The app will allow identifying patient’s needs, including unexpected needs, and providing patient-specific advice and decision support. It will include a feedback loop where patients can report their experiences and receive feedback in plain language via an external large language model. This interaction will then be analyzed to fine-tune the model continuously.
- Beyond a personalized medical assistant, the app may help discover unknown adverse effects of new treatments, making it a research tool for medical professionals.

Our team includes partners with experience in building AI systems, telemedicine, and psychiatry. A patients’ association will help to obtain access to thousands of questionnaires on patients’ needs for training the AI system.

### AI-Driven Drug Design

In the wake of the COVID-19 pandemic, this project emerges as a beacon of innovation, utilizing the power of generative AI to combat viral threats. The project employs advanced computational techniques to map out the complex protein-protein and protein-RNA interactions that define the viral lifecycle. By feeding these data into a deep learning model, this project aspires to transform raw biological data into actionable insights.

The project will refine the AI model through iterative training. Leveraging data from multiple international biobanks, the model will learn to discern new patterns that have eluded expert analysis. That paves the way for the predictive capabilities of the system, which is designed not only to understand the current strains of the virus but also to anticipate future mutations. This predictive model is a critical step towards preemptive drug design.

The generative AI system will extrapolate from known mutations to simulate possible future changes in the viral genome, creating a dataset of hypothetical virus variants. This dataset will provide an invaluable resource for preemptive drug design, enabling us to test and develop therapies against virus strains that have not yet emerged. This proactive approach is intended to shorten the response time in drug development when new strains are encountered, thereby mitigating potential public health crises with rapid therapeutic interventions.

Another pillar of the project is the integration of genetic data, where the AI model extends its analysis to the genetic predispositions that affect disease outcomes. Recognizing that the interplay of human and viral genetics can alter the course of an infection, the project will use genomic sequences from a diverse pool of patients. By training the model on this data, the project will uncover the genetic factors that contribute to the severity of the disease, leading to more personalized and effective treatments.

The project is committed to sharing its findings with the global scientific community. In its last phase, the team will create an open-access repository, making their data and AI model available to researchers worldwide. This collaborative approach not only accelerates the pace of discovery but also fosters a united front in the fight against pandemics. The project will also collaborate with pharmaceutical companies to prepare for possible drug screening and production in the future.

### Diagnostic Support and Prevention of Breast Cancer

This project will pioneer the integration of artificial intelligence in medical diagnostics across a consortium of European hospitals, via a decentralized approach enabling each participant to contribute to an AI model centrally hosted on a high-security cloud platform. Through this model, hospitals send updates derived from local MRI scan analyses in different countries, enhancing the model’s capability to detect early signs of breast cancer.

Crucially, this system allows for the pooling of vast amounts of diagnostic information without the actual transmission of raw patient data. In this setup, each hospital processes its own MRI data using local machine learning algorithms designed to identify predictive features indicative of breast cancer. This information is used to update the central model in real time. While the model learns from a growing pool of data points, individual patient data is neither seen nor stored by the central system, minimizing privacy risks. The project uses encryption and cybersecurity measures, ensuring that data transmitted to the central server remains shielded from potential breaches.

The size of the project’s distributed database will exceed all previous studies. The AI model will reach expert-level performance for breast cancer screening to prove the clinical benefit of distributed learning in terms of accelerated development and increased performance, to ultimately save thousands of lives.

The project targets the development of a robust industrial prototype with a view to developing a commercial product in the future. Medical doctors across Europe will interact with the AI system through a secure digital platform. Each doctor will upload MRI scans, and the system will instantaneously analyze the data to identify potential early signs of breast cancer. The doctors will receive a diagnostic report that highlights areas of concern. The output will be presented in the standard format of radiological reports, ready to be shared with the oncologist. This method aims to enhance the doctors’ ability to make informed decisions quickly, potentially increasing the accuracy of early cancer detection and treatment.
